# Do Black and Asian individuals wait longer for treatment? A survival analysis investigating the effect of ethnicity on time-to-clinic and time-to-treatment for diabetic eye disease

**DOI:** 10.1007/s00125-020-05364-5

**Published:** 2021-01-26

**Authors:** Varo Kirthi, Kate I. Reed, Ramith Gunawardena, Komeil Alattar, Catey Bunce, Timothy L. Jackson

**Affiliations:** 1 Faculty of Life Sciences and Medicine, King’s College London, London, UK; 2 King’s College Hospital NHS Foundation Trust, London, UK

**Keywords:** Clinical diabetes, Healthcare delivery, Retinopathy, Socioeconomic aspects

## Abstract

**Aims/hypothesis:**

This study explored the impact of ethnicity on time-to-clinic, time-to-treatment and rates of vision loss in people referred to hospital with diabetic eye disease.

**Methods:**

A survival analysis was performed on all referrals from an inner-city diabetic eye screening programme to a tertiary hospital eye service between 1 October 2013 and 31 December 2017. Exclusion criteria were failure to attend hospital, distance visual acuity in both eyes too low to quantify with the Early Treatment Diabetic Retinopathy Study (ETDRS) letter chart and treatment received prior to referral. Demographic and screening grade data were collected at the point of referral. Small-area statistics and census data were used to calculate indices of multiple deprivation. The main outcome measures were time taken from the date of referral for an individual to achieve the following: (1) attend the first hospital clinic appointment; (2) receive the first macular laser, intravitreal anti-vascular endothelial growth factor injection or pan-retinal photocoagulation treatment, in either eye; and (3) lose at least ten ETDRS letters of distance visual acuity, in either eye.

**Results:**

Of 2062 referrals, 1676 individuals were included. Mean age (± SD) was 57.6 ± 14.7 years, with 52% male sex and 86% with type 2 diabetes. The ethnicity profile was 52% Black, 30% White, 10% Asian and 9% mixed/other, with similar disease severity at the time of referral. Time-to-clinic was significantly longer for Asian people than for Black people (*p* = 0.03) or White people (*p* = 0.001). Time-to-treatment was significantly longer for Black people than for White people (*p* = 0.02). Social deprivation did not significantly influence time-to-treatment. There were no significant differences in the rates of vision loss between ethnic groups.

**Conclusions/interpretation:**

Black people wait longer for hospital eye treatment compared with their White counterparts. The reasons for this delay in treatment warrant further investigation.

**Graphical abstract:**

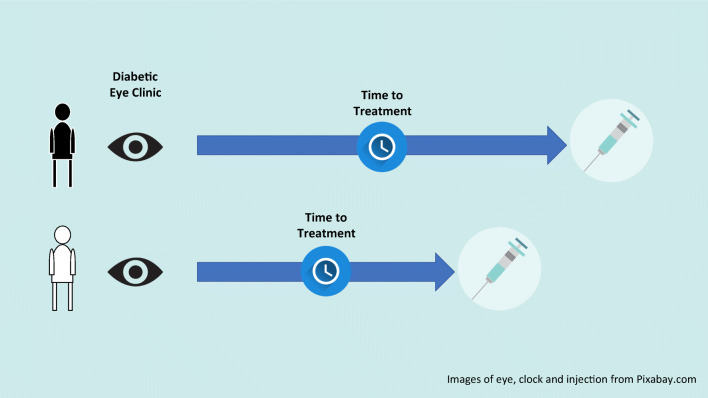

**Supplementary Information:**

The online version of this article (10.1007/s00125-020-05364-5) contains peer-reviewed but unedited supplementary material.

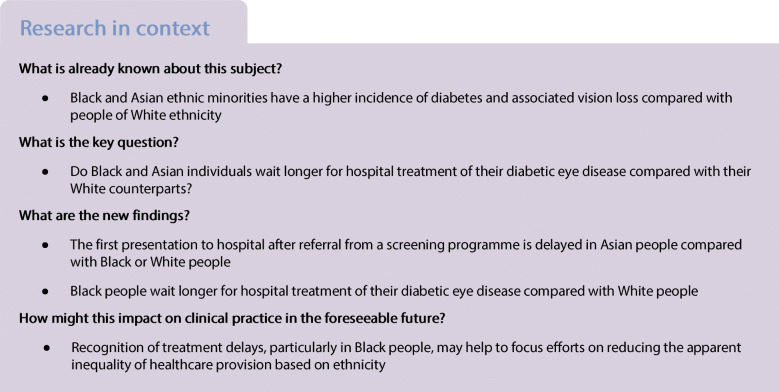



## Introduction

Diabetic retinopathy remains one of the principal causes of vision loss in adults of working age in developed countries, with a considerable health and socioeconomic impact [1]. A systematic review of 35 population-based studies of people with diabetes reported the prevalence of diabetic retinopathy, proliferative diabetic retinopathy (PDR), vision-threatening diabetic retinopathy and diabetic macular oedema (DMO) as 35%, 7%, 10% and 7%, respectively, although the actual rates varied widely among different ethnic groups [2]. In the UK, the incidence of diabetes is six times higher in people of South Asian origin and up to three times higher in people of African and African Caribbean origin [3]. Previous studies have also identified a higher prevalence of diabetic retinopathy and DMO in people of ethnic Black and Hispanic descent compared with people of White descent [4–7]. It is estimated that up to 95% of vision loss in diabetes is preventable or treatable, if detected early. Hence there is a pressing need to identify at-risk individuals [8].

There are also clinical and wider concerns that individuals from ethnic minority groups, particularly those of Black ethnicity, are treated differently in healthcare settings. Black individuals experience lower quality pain management, delays in receiving care within the Emergency Department, disparities in accessing cardiac tests, diagnoses and procedures, and are less likely to be placed on renal transplant waiting lists compared with their White counterparts [9–12]. Reasons for this variation are complex and multifactorial but remain poorly understood.

There is also a paucity of data on whether treatment is delayed in ethnic minority groups, and how this impacts health outcomes. The aim of this study was to explore ethnic variation within a metropolitan population by testing time-to-clinic, time-to-treatment and rates of vision loss in individuals referred to hospital with a similar severity of diabetic eye disease.

## Methods

### Ethics statement

This study adhered to the revised Declaration of Helsinki (2008). After review by the King’s College Hospital NHS Foundation Trust Research & Innovation and Audit departments, this study was deemed a service evaluation. Written consent from patients was not required as only anonymised or pseudo-anonymised data were analysed.

### Patient and public involvement

Patients have been invited to help interpret the results of this study and develop a dissemination strategy.

### Design, setting and population

Data were collected from the diabetic eye clinic at King’s College Hospital NHS Foundation Trust, a tertiary centre that cares for a diverse population of African, African Caribbean and Asian ethnic groups in South-East London, an area with a high prevalence of social deprivation. Healthcare professionals working within the eye clinic were from a variety of ethnic backgrounds; however, most ophthalmologists were of White ethnicity and none were of Black ethnicity. A survival analysis was performed on all referrals from the South-East London Diabetic Eye Screening Programme (DESP) to the hospital for routine (<6 weeks) or urgent (<2 weeks) hospital eye clinic review in accordance with UK National Screening Committee (NSC) guidelines, between 1 October 2013 and 31 December 2017 [13]. Data on hospital eye clinic outcomes were collected up until 31 December 2019.

### Eligibility criteria

Screening in the UK is offered annually to any individual with diabetes aged 12 years or over [13, 14]. No restrictions were placed on the age, sex, ethnicity, type of diabetes, visual acuity or grade of retinopathy of individuals referred from DESP for hospital eye clinic review. Exclusion criteria were as follows: (1) failure to attend hospital; (2) distance visual acuity in both eyes too low to quantify with Early Treatment Diabetic Retinopathy Study (ETDRS) letters; and (3) treatment received prior to the date of referral. Individuals exempt from DESP, such as those with no perception of light or those already under the care of the hospital eye clinic due to sight-threatening disease or because the fundus cannot be assessed by digital photography, were also excluded. Individuals with a sight-threatening disease of non-diabetic aetiology (e.g. glaucoma), but still remaining under the care of DESP, were not excluded from analysis.

### Outcomes

The main outcomes of interest were as follows: (1) time-to-clinic, (2) time-to-treatment and (3) rate of vision loss. Time-to-clinic was measured from the date of the initial DESP referral to the date of the first hospital clinic review. Time-to-treatment was measured from the date of the initial DESP referral to the date when any of the following events were first recorded in hospital, in either eye:

1. Macular focal or grid laser treatment for DMO
2. Intravitreal anti-vascular endothelial growth factor (anti-VEGF) treatment for DMO
3. Pan-retinal photocoagulation (PRP) laser treatment for PDR

Rate of vision loss was defined as the time taken to lose at least ten ETDRS letters of distance visual acuity in either eye, from the baseline vision recorded at the first hospital eye clinic appointment after DESP referral. The minimum threshold for vision loss was set at ten ETDRS letters (0.2 logarithm of the minimum angle of resolution [logMAR]) based on published data on test–retest variability in real-world settings [15–18]. For refractive errors or emmetropia, best-corrected visual acuity (BCVA) was obtained by testing with usual distance glasses or unaided, respectively. Where BCVA was not recorded, unaided (UVA) or pinhole (PHVA) visual acuity was used instead. If only UVA was recorded at baseline, a significant visual acuity loss was only noted if a drop of at least ten ETDRS letters was observed using a more accurate method on a subsequent occasion, such as BCVA or PHVA. Snellen or logMAR visual acuities were converted to equivalent ETDRS letters [19].

### Data collection

The following demographic data were collected from DESP: (1) age; (2) ethnicity; (3) type of diabetes; (4) referral date; (5) type of referral (routine or urgent); and (6) screening grade in each eye as per NSC guidelines [13]. Data on hospital eye clinic outcomes were retrieved from electronic patient records (EPR) (Medisoft, Leeds, UK) and laser treatment logbooks. Patient administration systems were used to retrieve clinic dates for time-to-event analyses.

### Ethnicity data

Self-reported ethnicity was divided into four groups from the 16 national census categories in the UK (electronic supplementary material [ESM] Table 1) [20]. Anonymised pooled ethnicity data on individuals invited for annual diabetic retinopathy screening between 2013 and 2017 were obtained directly from DESP for baseline comparison. Population ethnicity data for Lambeth and Southwark, the two south-east London boroughs from which screened individuals are referred to King’s College Hospital, were also obtained from census data [20].

### Index of Multiple Deprivation data

Pseudo-anonymised data were used to extract the Lower-layer Super Output Area (LSOA) for each individual. LSOAs are a geographical hierarchy to improve the reporting of small-area statistics in England and Wales. Each LSOA is generated to cover approximately 1000–1500 residents. Data from each LSOA were used to extract Index of Multiple Deprivation (IMD) deciles for every individual from national census data in England [20]. IMD deciles were grouped into quintiles for statistical analysis.

### Statistical analysis

Data were analysed using STATA version 16 (StataCorp, College Station, TX, USA). Analyses were largely descriptive and included an analysis of diabetic retinopathy severity by ethnicity and IMD, at the point of referral. Continuous data were reported as means with SDs, if approximately normally distributed by inspection of histograms, or as medians and IQRs if marked non-normality was observed. Categorical data were reported as numbers and frequencies. Kaplan–Meier plots were constructed to examine the time from referral to the following events of interest: (1) first hospital clinic review; (2) first macular laser, intravitreal anti-VEGF or PRP laser treatment in either eye; and (3) distance visual acuity loss of at least ten ETDRS letters in either eye. The logrank test was used to assess the statistical significance of observed differences in treatment event rates between Black, White and Asian participants. Cox regression analysis was used to explore factors affecting time-to-treatment. Separate analyses were conducted by ethnicity and IMD, where data were sufficient.

## Results

**Fig. 1:**
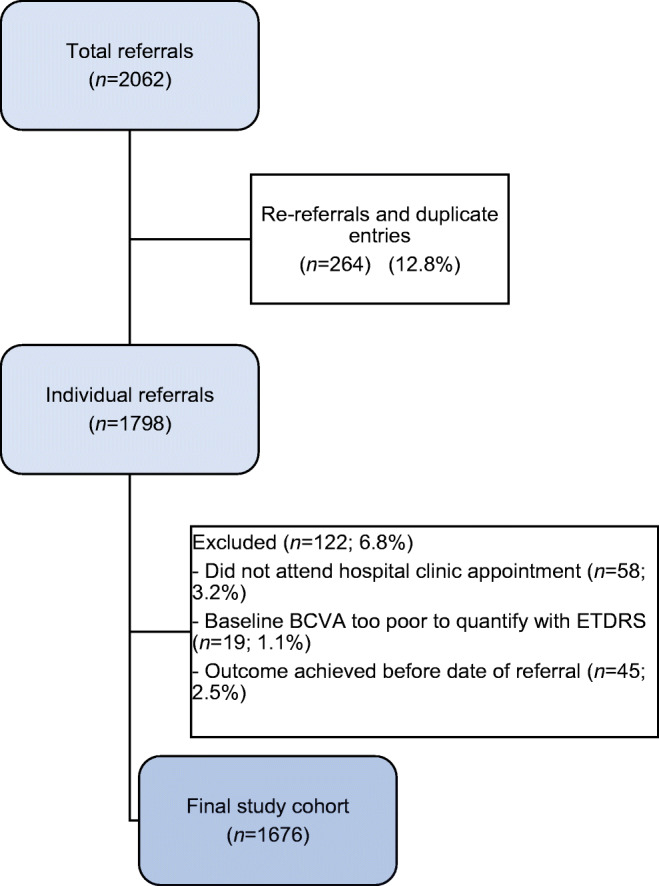
Flow chart outlining the process for selecting the study population

A total of 2062 referrals for 1798 people with diabetes were made from the screening service to the hospital diabetic eye clinic between 1 October 2013 and 31 December 2017. After applying the eligibility criteria, a final cohort of 1676 people remained. A flow chart outlining the reasons for exclusion is provided in Fig. 1.

### Baseline characteristics

Table 1 lists the baseline demographics of the study population. The mean age (± SD) of referrals was 57.6 ± 14.7 years, with a predominance of type 2 diabetes (*n* = 1448 [86%]). The vast majority of the study population were in the first five IMD deciles (*n* = 1500 [90%]); almost half (*n* = 759 [45%]) lived in LSOAs ranked within the 20% most deprived regions in England (ESM Table 2). Of those with the most severe diabetic retinopathy grading of R3A or M1, 48% and 46%, respectively, were from the two most deprived IMD deciles [13, 21].

**Table 1:** Baseline demographics of the study cohort

Characteristic	*n*	%
Total cohort	1676	–
Mean age (SD), years	57.6 (14.7)	–
Male sex	876	52.3
Type 1 diabetes	228	13.6
Type 2 diabetes	1448	86.4
Black	866	51.7
White	495	29.5
Asian	169	10.1
Mixed/other	146	8.7

Of the 58 individuals who did not attend (DNA) any hospital eye clinic appointments during the study period, 91% (53) were from the more deprived half of the population based on IMD and 43% (25) were from LSOAs ranked within the 20% most deprived regions in England. Regarding ethnicity, 52%, 21% and 19% of participants were Black, White and Asian, respectively. Compared with the baseline data for all referrals (Table 1), this suggests a higher than expected number of Asian people in the DNA cohort, but no association with IMD.

Table 2 lists the distribution of screening grades, according to ethnicity, in the worse eye at the point of referral. There were no significant differences between the ethnic groups in the distribution of referrals by urgency or screening grade. The majority of routine referrals were for M1 maculopathy, according to NSC grading definitions and referral guidelines [13, 21].

**Table 2:** Referral type and screening grade by ethnicity

Referral type or screening grade	Black	White	Asian	Mixed/other	Total number
Referral type					
Routine	835 (92)	464 (90)	170 (93)	136 (88)	1605
Urgent	74 (8)	53 (10)	12 (7)	18 (12)	157
Screening grade					
R0	1 (<1)	1 (<1)	1 (<1)	0 (<1)	3
R1	606 (70)	305 (62)	108 (64)	89 (61)	1108
R2	151 (17)	119 (24)	40 (24)	33 (23)	343
R3S	38 (4)	17 (3)	7 (4)	6 (4)	68
R3A	68 (8)	50 (10)	12 (7)	17 (12)	147
M0	60 (7)	69 (14)	21 (12)	18 (12)	168
M1	804 (93)	423 (86)	147 (88)	127 (88)	1501
Ungradable in both eyes	2 (<1)	3 (<1)	1 (<1)	1 (<1)	7

Data are presented as *n* (%). Percentages are based on the total number of participants within each ethnic group

Screening grade is reported for the worse eye at the point of referral

M, maculopathy; R, retinopathy

### Ethnicity profiles

The ethnicity profiles of the study population were compared with the diabetes screening population (data from DESP 2018) and the general population in the London Boroughs of Lambeth and Southwark (data from 2011 national census) [20, 22]. While the proportion of White and Asian people was similar when comparing the screening population (31% and 10%, respectively) and those referred to hospital (30% and 10%, respectively), Black people were over-represented in the referral cohort (ESM Table 3). Compared with the general population, Black people were represented twice as frequently among those referred to hospital (52% vs 26%).

### Event rates

From the 1676 individuals included, 280 (17%) underwent a therapeutic intervention while 411 (25%) experienced a visual acuity loss of at least ten ETDRS letters in at least one eye during the study period. There were no significant differences in the frequency of observed events among Black, White and Asian ethnic groups, as determined by χ^2^ tests (ESM Table 4).

### Time-to-clinic

The time taken from referral to the date of the first hospital eye clinic review was compared by ethnicity for all 1798 referrals. A logrank test revealed a significantly longer time to first clinic review after referral in Asian people compared with either Black (χ^2^ = 4.70, *p* = 0.03) or White (χ^2^ = 10.58, *p* = 0.001) people. Although time-to-clinic was longer in Black compared with White people, this did not reach statistical significance (χ^2^ = 3.45, *p* = 0.06).

### Time-to-treatment

**Fig. 2:**
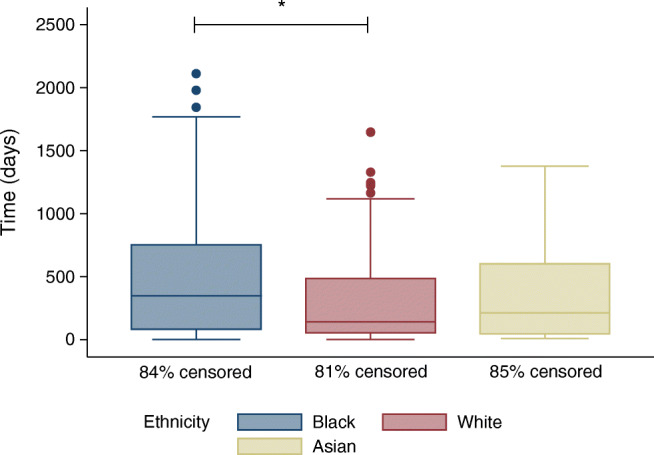
Boxplots comparing time-to-treatment. Boxplots show the time in days to first macular laser, intravitreal anti-VEGF or PRP laser treatment in either eye, by ethnicity. The central line is the median, the edges of the boxes are Q1 (25th percentile) and Q3 (75th percentile). The ends of the whiskers are the upper and lower adjacent values, which are the most extreme values within Q3 + 1.5(Q3−Q1) and Q1 − 1.5(Q3−Q1), respectively. All circles outside these whiskers represent outliers. *p<0.05 for difference between the groups. Censor rates are displayed by ethnicity

**Fig. 3:**
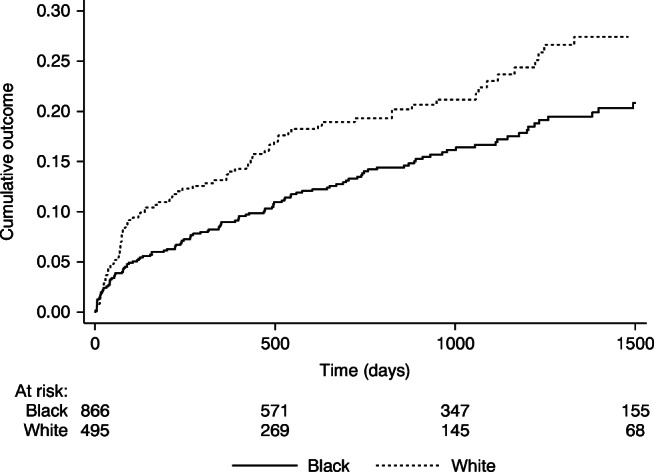
Kaplan–Meier failure analysis of time-to-treatment. Survival analysis of time to first macular laser, intravitreal anti-VEGF or PRP laser treatment in either eye, comparing Black and White people. Numbers at risk are shown by ethnicity for specific time points. *p*=0.02 by logrank test

Figure 2 shows the time-to-treatment (first macular laser, intravitreal anti-VEGF or PRP laser treatment in either eye) by ethnicity. A logrank test revealed a significantly longer time to macular laser, intravitreal anti-VEGF or PRP laser treatments in Black people compared with White people (χ^2^ = 5.67, *p* = 0.02), shown by the Kaplan–Meier analysis in Fig. 3. There was no significant difference in time-to-treatment between Asian people and their White (χ^2^ = 1.84, *p* = 0.17) or Black (χ^2^ = 0, *p* = 0.99) counterparts. To explore whether these differences in time-to-treatment might be driven by a delay in attending the first hospital appointment, rather than a delayed offer or agreement to undergo treatment once in the hospital system, a post hoc analysis was performed by recalculating time-to-treatment from the date of first hospital attendance. This analysis confirmed a longer time-to-treatment in Black compared with White people (χ^2^ = 5.14, *p* = 0.02), with no significant differences between Asian and either White (χ^2^ = 2.85, *p* = 0.09) or Black people (χ^2^ = 0.27, *p* = 0.6).

### Rate of vision loss

**Fig. 4:**
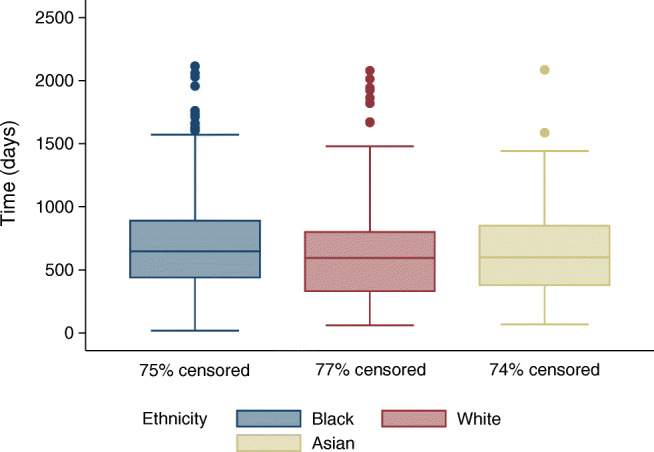
Boxplots comparing rates of vision loss. Boxplots show the time taken in days to achieve a drop of at least ten letters in distance visual acuity in either eye, by ethnicity, from the baseline vision recorded at the first hospital appointment. The central line is the median, the edges of the boxes are Q1 (25th percentile) and Q3 (75th percentile). The ends of the whiskers are the upper and lower adjacent values, which are the most extreme values within Q3 + 1.5(Q3−Q1) and Q1 − 1.5(Q3−Q1), respectively. All circles outside these whiskers represent outliers. Censor rates are displayed by ethnicity

Figure 4 shows the time taken in days to achieve a drop of at least ten letters in distance visual acuity in either eye, from the baseline vision recorded at the first hospital appointment. A logrank test for equality of survivor functions did not reveal any significant differences in time taken to achieve this level of vision loss between Black, White and Asian people.

### Social deprivation

Given that 45% of individuals were within the most deprived IMD quintile, survivor function in this quintile was compared with the other quintiles. A logrank test did not reveal any significant differences between the most deprived IMD quintile and all other IMD quintiles, for both time-to-treatment (χ^2^ = 0.02, *p* = 0.89) and time to visual acuity loss of ten or more ETDRS letters (χ^2^ = 0.54, *p* = 0.46).

### Regression analysis

Cox regression analysis was used to explore the effects of diabetes type, sex, ethnicity, severity of retinopathy, presence of maculopathy and IMD quintile on the likelihood of receiving macular laser, anti-VEGF and PRP laser interventions at any given point in time. Male sex was associated with a significantly increased HR compared with female sex, while Black ethnicity was associated with a significantly reduced HR compared with White ethnicity, as shown in Table 3.

**Table 3:** Factors affecting the intervention rate, using the Cox proportional hazards model

Factor	Events (*n*)	HR (95% CI)	HR (95% CI) adjusted for age
Diabetes type			
Type 1	44	–	–
Type 2	236	0.76 (0.55, 1.05)	0.92 (0.58, 1.44)
Sex			
Female	118	–	–
Male	162	1.31 (1.03, 1.66)*	1.34 (1.05, 1.72)*
Ethnicity			
White	92	–	–
Black	137	0.73 (0.56, 0.95)*	0.71 (0.53, 0.95)*
Asian	26	0.73 (0.47, 1.13)	0.78 (0.50, 1.24)
Retinopathy grades R1/2			
White	50	–	–
Black	89	0.85 (0.60, 1.20)	0.80 (0.55, 1.18)
Asian	16	0.78 (0.44, 1.37)	0.77 (0.42, 1.38)
Retinopathy grades R3S/A			
White	42	–	–
Black	48	0.58 (0.38, 0.88)*	0.80 (0.44, 1.44)
Asian	10	0.87 (0.44, 1.74)	1.11 (0.46, 2.72)
Maculopathy			
M0	30	–	–
M1	250	0.96 (0.65, 1.40)	0.93 (0.62, 1.38)
IMD quintile			
1	129	–	–
2	88	0.89 (0.68, 1.16)	0.92 (0.69, 1.22)
3	44	1.17 (0.83, 1.64)	1.21 (0.84, 1.75)
4	15	1.22 (0.72, 2.09)	1.21 (0.69, 2.12)
5	4	0.94 (0.35, 2.55)	0.90 (0.32, 2.53)

Hazard ratios (HR) are presented with 95% CIs and also adjusted for age as a continuous variable

The ‘hazard’ was any macular laser, anti-VEGF or PRP laser treatment event

*p<0.05, compared with the first subgroup listed under each factor

## Discussion

Despite a similar burden of disease at referral that might require treatment (Black vs White people, respectively: 8% and 10% at retinopathy grade R3A; 93% and 86% at maculopathy grade M1), time-to-treatment was significantly longer in Black people than in White people. Black people did ultimately have similar treatment rates, but these treatments took longer to occur. However, no association was found between social deprivation and time-to-treatment.

Similarly, despite a similar distribution of routine and urgent referrals, the time to first hospital eye clinic review, from referral, was longer in Asian people compared with their Black or White counterparts. A longer time-to-clinic could be a contributory factor to the reported sixfold higher incidence of diabetic retinopathy in Asian vs non-Asian populations [3]. Unlike their Black counterparts, there was no significant difference in time-to-treatment between Asian and White people. A longer time-to-clinic may result in a greater severity of disease at first presentation, which could lead to expedited treatment. This requires further exploration but might explain why time-to-treatment may not have been significantly delayed in Asian people.

Previous studies have shown that minority ethnic groups are twice as likely to have sight-threatening diabetic retinopathy, with South Asians being three times more likely than White people to be registered blind due to diabetic retinopathy [23–25]. In this study, ≥10 ETDRS letters was chosen as the minimum threshold for a clinically important vision loss to the individual, while also minimising any confounding generated by test–retest variability. Despite disparities in time-to-clinic and time-to-treatment, there was no statistically significant difference in the rates of vision loss among Black, White and Asian people. However, the proportion of individuals who reached a ≥10 ETDRS letter drop was lower in White individuals (112/495, 23%) compared with their Black (213/866, 25%) and Asian (44/169, 26%) counterparts (ESM Table 4). While these differences are small and difficult to interpret in low numbers, they do suggest that ethnic minorities may be experiencing higher rates of vision loss, in keeping with previously published reports.

The number of individuals who DNA any hospital eye clinic appointment during the study period was only 58 (3%) of the total number of referrals (1798). This is likely to be an underestimate of the total DNA rate, as some individuals may only have attended the hospital after re-invitation to clinic or even re-referral. However, in order to maximise the sample size, individuals who had failed to attend at least one clinic appointment were not excluded from the analysis. Of interest, White people were underrepresented in this DNA cohort compared with other ethnic groups, especially Asians.

Several factors are known to increase hospital non-attendance and loss to follow-up including ethnicity, increasing age, reduced mobility, poor baseline vision and fear of the clinical outcome [26–29]. Language and cultural barriers may also play an important role, particularly among South Asians, in whom work and family commitments are reported to be prioritised over health-seeking behaviours and such factors might contribute to a delayed initial presentation to clinic [30]. While a long distance between home and the hospital is a recognised factor, this is unlikely to have had a significant impact in this study, given that the DESP clinic is co-located within the main hospital eye clinic.

The burden of comorbid diseases and disability may also influence attendance at clinic and treatment appointments. In the USA, Black people are almost four times as likely to develop renal failure than their White counterparts [31]. Despite only constituting around 13% of the general population, 35% of all patients receiving dialysis for renal failure are of Black origin [31]. Black people not only experience a higher prevalence of type 2 diabetes but also suffer from a disproportionate burden of serious complications, such as hypertension and stroke, with associated disabilities, compared with White people [32]. Competing medical appointments, hospitalisations and transport difficulties could all potentially hinder timely treatment of eye disease.

Variation in the provision of healthcare is widely reported among minority ethnic groups but is poorly understood [9–12]. Reported factors include a lack of engagement with healthcare providers and physician bias. Higher levels of physician distrust have also been reported among male patients and those from ethnic minorities or low socioeconomic backgrounds [33, 34]. A lack of perceived benefit, particularly in the absence of any visual symptoms in early diabetic eye disease, may also contribute to reduced engagement with hospital eye services and a reluctance to accept treatment, such as laser or intravitreal injections.

Racial stereotypes and unconscious bias are known to influence physician behaviour towards minority ethnic groups, particularly with respect to treatment choices offered [35–37]. A recent study of over 80,000 patients with type 2 diabetes in England found that Black patients were 50% less likely and Asian patients 15% less likely to be prescribed newer medications such as sodium–glucose cotransporter 2 inhibitors and glucagon-like peptide-1 agonists, compared with their White counterparts [38]. Similarly, Black people with age-related macular degeneration (AMD) were found to be 23% less likely to receive anti-VEGF treatment and 18% less likely to have regular eye examinations for AMD compared with White people [39].

Referrer bias in our study is unlikely as the DESP fundus image graders did not have ethnicity data readily available, and high numbers of Black people were referred to the hospital service. In face-to-face clinic reviews, physician bias is possible and this could be one possible reason for the delayed time-to-treatment seen in Black people; however, this is hard to confirm or quantify.

Limitations of this study include an over-representation of individuals from areas of social deprivation, constraining analysis of the impact of social deprivation on time-to-treatment. As only small numbers of Asian and other non-Black minority ethnic groups were present in this study, we could not explore specific at-risk subgroups, such as people of South Asian and Hispanic ethnicity. A lack of refracted BCVA in all visits may reduce the accuracy of visual acuity measurements but it might be expected to affect all groups similarly and PHVA usually overcomes any uncorrected refractive error. The cause of the fall in visual acuity also could not be determined and while it is reasonable to assume this was most often due to diabetic eye disease, other conditions could co-exist, such as glaucoma and AMD, which are known to vary among ethnic groups [40]. Only individuals who attend annual eye screening were included in this study but this accounts for 81.5% of the total diabetic population living within the London Boroughs of Lambeth and Southwark [22]. Data collection was retrospective but that may be helpful when studying patient and clinician behaviour, as prospective data collection might influence behaviour. No data were collected on those already under the care of hospital eye services and those excluded from annual eye screening due to very low vision. Hence, the true prevalence of low vision among individuals with diabetes is likely to be greater, as previously reported [25].

In summary, despite similar disease severity at the point of referral, this study suggests that Black people were either less likely to be offered treatment in a timely manner or were less likely or unable to accept an offer of treatment. This warrants further investigation and future studies could consider gathering the following qualitative data from individuals with diabetes: (1) their knowledge of the underlying health condition; (2) their beliefs surrounding treatments for diabetic eye disease; and (3) their experience of interactions with ophthalmologists in hospital. It would also be helpful to know if the ethnicity of a patient somehow influences the clinical approach of the attending ophthalmologist. Improved understanding may reduce the apparent inequality of healthcare provision based on a patient’s ethnicity.

## Supplementary information

ESM (PDF 158 kb)

## Data Availability

The datasets generated during and/or analysed during the current study are available from the corresponding author on reasonable request.

## Abbreviations

AMD: Age-related macular degeneration
BCVA: Best-corrected visual acuity
DESP: Diabetic Eye Screening Programme
DMO: Diabetic macular oedema
DNA: Did not attend
ETDRS: Early Treatment Diabetic Retinopathy Study
IMD: Index of Multiple Deprivation
LSOA: Lower-layer Super Output Area
NSC: National Screening Committee (UK)
PDR: Proliferative diabetic retinopathy
PHVA: Pinhole visual acuity
PRP: Pan-retinal photocoagulation
UVA: Unaided visual acuity
VEGF: Vascular endothelial growth factor
