# Supplementary material for: Do Black and Asian individuals wait longer for treatment? A survival analysis investigating the effect of ethnicity on time-to-clinic and time-to-treatment for diabetic eye disease
Source: Diabetologia. 2021 Jan 26;64(4):749–57. doi: 10.1007/s00125-020-05364-5 (PMC7940160; doi:10.1007/s00125-020-05364-5)
Supplement: Supplementary file 1 — (PDF 158 kb) [file 125_2020_5364_MOESM1_ESM.pdf]

ELECTRONIC SUPPLEMENTARY MATERIAL

ESM Table 1

Ethnicity groups by census classification. Definitions of ethnicity groups used in this study and corresponding UK census categories.

Ethnicity group	Census categories included
White	British, Irish, any other White background
Black	Caribbean, African, any other Black background
Asian	Indian, Pakistani, Bangladeshi, Chinese, any other Asian background
Mixed/Other	White and Black Caribbean, White and Black African, White and Asian, any other Mixed background, any other Ethnic group

ESM Table 2

Indices of multiple deprivation (IMD). Indices of multiple deprivation calculated by comparing Lower-layer Super Output Areas to census data in England and placed in deciles.

IMD decile by LSOA	*n*	%
1 (most deprived area)	108	6.4
2	651	38.8
3	349	20.8
4	235	14.0
5	157	9.4
6	77	4.6
7	41	2.4
8	33	2.0
9	20	1.2
10 (least deprived area)	5	0.3

IMD, index of multiple deprivation; LSOA, Lower-layer Super Output Area

ESM Table 3

Comparison of the ethnicity profiles by population. Comparison of the general population of the London Boroughs of Lambeth and Southwark with the South-East London DESP screening population and study population. * Obtained from 2011 national census (20). ** Obtained from 2018 DESP audit (22).

**Ethnicity**	**General population of Lambeth & Southwark***		**DESP screening population****		**Referred to hospital (study population)**	
	n	%	*n*	%	*n*	%
Black	156,053	**26**	6655	**44**	866	**52**
White	329,374	**56**	4675	**31**	495	**30**
Asian	48,130	**8**	1521	**10**	169	**10**
Mixed/Other	57,812	**10**	1089	**7**	146	**9**
Not Declared	-	-	1038	**7**	-	-
**Total**	**591,369**		**14,978**		**1676**	

DESP, Diabetic Eye Screening Programme

ESM Table 4

Comparison of event rates by ethnicity. Pearson's chi-squared test probabilities showing no significant difference in observed frequencies by ethnicity.

**Event recorded at least once in either eye during the study period**	**Ethnicity**						**Pearson's chi-squared test probability**
	**Black**		**White**		**Asian**		
	n	%	*n*	%	*n*	%	
Macular laser	53	**6**	29	**6**	8	**5**	0.983
Anti-VEGF	66	**8**	37	**7**	12	**7**	0.972
PRP laser	65	**8**	55	**11**	12	**7**	0.066
VA loss of ≥10 letters	213	**25**	112	**23**	44	**26**	0.592
Total participants	866		495		169		-

PRP, pan-retinal photocoagulation; VA, visual acuity; VEGF, vascular endothelial growth factor
